# Chronic Low Back Pain Forced Me to Search for and Find Pain Solutions: An Autobiographical Case Report

**DOI:** 10.7759/cureus.21529

**Published:** 2022-01-23

**Authors:** Helene Bertrand

**Affiliations:** 1 Department of Family Practice, University of British Columbia, North Vancouver, CAN

**Keywords:** chronic low back pain (clbp), chronic nonspecific low back pain, treating low back pain, topical pain relief, diabetic neuropathic pain, undestanding and treating pain, sacroiliac joint dysfunctional pain, sacroiliac, holistic pain management, chronic and acute pain management

## Abstract

Lifelong, pregnancy-induced low back pain forced me to search for solutions to the problem of pain. Currently, low back pain is often diagnosed as “nonspecific” and, as a result, a multitude of tests and poorly effective, at times side effect-laden or habit-forming treatments are recommended. My quest for relief took me to first diagnose my pain as coming from the sacroiliac joints, then to prolotherapy, the first treatment which brought me prolonged relief. I then learned how to perform prolotherapy. In 2009, when I undertook a randomized controlled study of dextrose prolotherapy for rotator cuff tendinopathy, I restricted my practice to treating pain.

As low back pain was a large part of my practice, I sought new ways to examine the sacroiliac joints. I conducted a consecutive patient data collection which suggested that over three-quarters of those with low back pain suffer from displaced sacroiliac joints. In a further randomized controlled study, I found that the two-minute corrective exercise I derived from this test provided immediate relief to 90% of those using it.

With Dr. John Clark Lyftogt I discovered the safety and effectiveness of 5% dextrose perineural injections to provide immediate pain relief to any area supplied by a nerve I could reach with my needle. As I was treating many diabetics with peripheral neuropathy, I shifted my perineural injection material to 5% mannitol, which may be as effective, with less exposure to dextrose as a potential benefit for diabetics.

As most people dislike injections, a pharmacist and I developed a mannitol-containing topical cream for pain relief. We compared a base cream to the same cream with mannitol on lips pretreated with capsaicin cream which made them burn. By 10 minutes the probability the two creams were as effective in relieving the burn was less than 0.001 in favor of mannitol. When given to 235 patients with a total of 289 different painful conditions, we found that it provided 53% relief in an average of 16 minutes with a median of four hours duration.

Now retired, after 55 years of medical practice, I love to relieve the pain of friends and fellow hikers using exercise and cream. Searching for and finding solutions to chronic pain has enriched my life and that of many others.

## Introduction

Chronic pain afflicts one person in five [1]. After prescribing a multitude of different treatments and sending them for a number of consultations, I often felt stymied and frustrated at being unable to help these patients, at times even harming them with side effects or addiction. The most disabling of these was low back pain, a condition I was personally familiar with [2].

This is a story about my 37 years of low back pain, how I reached a diagnosis, found effective treatments, tried these on my patients, then showed through research that they worked. I hope those who read this will learn, as I did, the poorly known yet effective treatments they can apply to relieve many of the painful conditions which afflict their patients.

## Case presentation

It was June 1966, in the last weeks of my rotating internship, at the end of my first pregnancy. Straightening out after helping a patient, I felt a sudden, piercing, excruciating pain in my lower back, which forced me to remain partially bent forward until the end of my pregnancy. After I delivered my baby, I had trouble picking her up and walking with her. I carried her upstairs by sitting on the steps and pushing myself up from one step to the next.

The physiatrist I consulted gave me a diagnosis of nonspecific low back pain and referred me for physiotherapy. I was treated with back traction as well as exercises and told to stay active. The back pain lasted several months then subsided, only to recur frequently. It got worse with my subsequent pregnancies and would flare up when I tried to pick up one of my three children.

In 2003, I was attending a lecture on the diabetic foot with a friend of mine, Dr. Murray Allen, who was a physical medicine specialist. As we sat next to each other, I complained about my sacroiliac joint pain. Whereupon he offered to treat me with prolotherapy. I had no idea what prolotherapy was. He explained that he would inject the periosteal insertions of the ligaments stabilizing my sacroiliac joints with a 25% dextrose solution which would create a small injury leading to inflammation. The inflammation would stimulate the growth of small vessels from the richly vascularized periosteum into the very poorly vascularized ligaments. These vessels would carry fibroblasts into the damaged ligaments. The fibroblasts would deposit collagen there, forming scar tissue to strengthen these stabilizing ligaments.

After 37 years of severe recurrent low back pain, I was ready to try anything. A few nights later, I went to his house and lay prone as he treated me with multiple injections in my low back. The procedure was so painful that I was forced to do my labor breathing exercises throughout. The next few days were uncomfortable but gradually my back felt better. I repeated the prolotherapy treatment twice, one month apart, and subsequently became pain-free for almost one year.

Having experienced such relief, I started referring many of my patients with musculoskeletal pain to Dr. Allen. Most of them came back to the office reporting on how much better they felt. Unfortunately, six months after he had treated me, Dr. Allen told me he was retiring. I was concerned that my patients and I would no longer have access to this very helpful therapy, so he offered to train me. As I was off every Friday, he came to my office and supervised me as I treated anywhere between three and 10 of my patients using 25% dextrose prolotherapy injections. I started taking courses on how to perform prolotherapy and attended the meetings of the American Association for Orthopedic Medicine (AAOM), the Hackett Hemwall Patterson Foundation (HHPF), and, later, the Canadian Association for Orthopedic Medicine (CAOM).

Now that I knew how effective prolotherapy was in relieving musculoskeletal pain, I would always include one vial of 25% dextrose, some 10 mL syringes with special pushers I invented, alcohol swabs, and #30G 1-inch needles wherever I was traveling. Using smaller needles with a pusher makes the procedure much more comfortable, similar to acupuncture, but able to stimulate repair.

In 2008, while sailing on the Mediterranean, I developed a painful and restricting rotator cuff tendinopathy. Fortunately, I was able to treat myself with prolotherapy: I inserted the needles in the various tendon insertions on the periosteum, where I was tender, and asked my husband to push in 1 mL of solution in each location. One week later, I was feeling much better.

Meanwhile, about once a year, my back pain would recur, and one of my assistants would treat me with prolotherapy. Usually, a single session would suffice. The problem was that, depending on where I was or what I was doing, prolotherapy was not always available, and I could not reach that area of my back to treat myself. Also, many of my low back pain patients could not afford the cost of prolotherapy, so I started to search for other ways to treat severe low back pain from sacroiliac displacement.

In the early 60s, when I went to medical school, our main diagnostic instruments were our ears, our eyes, and our fingers. Medical imaging consisted of x-rays: no ultrasounds, CT scans, or MRIs. We were trained in palpation. If someone had pain, we were taught to put our finger where it hurt, and we knew what anatomical structure we were pressing on. We reached a diagnosis when pressure on a structure caused pain.

Finding that my sacroiliac joints were tender to pressure on the long dorsal sacroiliac ligaments just below the PSISs, I hypothesized that the pain was caused by the nerves in those ligaments being overstretched by a displaced ileum. To reposition the ileum, I would need to determine to what degree and in which direction the innominate bone had been displaced; in other words, I would need to measure the relative heights of the posterior superior iliac spines (PSISs). In 1999, Levangie described how she compared the distance from the floor to each of the PSISs to determine whether one of them was displaced, and in what direction [3]. Unfortunately, I lacked the equipment to take such measurements.

Perhaps inspired by my father, brother, and sister, all engineers, I intuited that it is not the distance from the floor that matters, but rather just the difference between the heights of each PSIS joint; instead of using a floor-based measuring instrument, I could just use a carpenter’s level. By 2014, I had developed the sacroiliac forward flexion test (SIFFT) where I compared the levels of the PSISs and measured the distance between the two levels. Levangie’s work encouraged me to use a 5 mm cut-off for diagnosing sacroiliac joint asymmetry [3]. If the PSIS on the painful side was higher, the ileum was displaced anteriorly. To straighten this out, it seemed logical to force the ileum backward with the thigh pushing on the anterior superior iliac spine (ASIS). If the PSIS on the painful side was lower than the other, the ileum was displaced posteriorly. It could be pulled forward by hyperextending the thigh so that the sartorius and the rectus femoris could pull on the ASIS and the anterior inferior iliac spine (AIIS). I devised some two-minute exercises to achieve this and called them the sacroiliac forward flexion test and corrective exercise (SIFFT-E).

**Figure 1:**
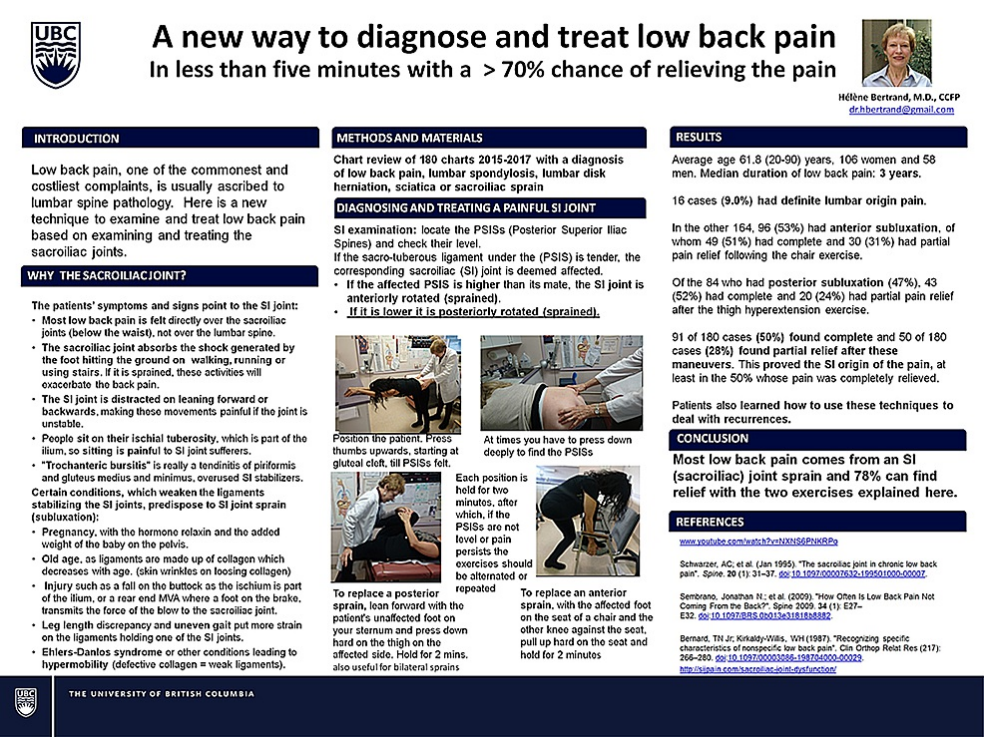
A new way to diagnose and treat low back pain in <5 minutes with >70% chance of relieving the pain Poster presentation #119, Canadian pain Society meeting May 24, 2018. A total of 180 charts (2015-2017) were reviewed. If the sacrotuberous ligament under a PSIS was tender, the corresponding SI joint was deemed affected. If the affected PSIS was higher than its mate, the SI joint was anteriorly subluxated. If it was lower, it was posteriorly subluxated. Anterior subluxation was corrected by jamming the thigh on the affected side against the anterior superior iliac spine (ASIS) using a chair. Posterior subluxation correction involved hyperextending the thigh on the affected side. Each exercise was held for two minutes. The chair exercise was strenuous, and the examining table treatment required an assistant. This prompted the development of the stretch exercise to allow low back pain sufferers a way to self-relieve their pain at all times. Exact measurement of the distance between the right and left PSIS levels was not taken. To measure the patients’ exercise effectiveness and their progress in leveling the PSISs, the SIFFT was developed. SIFFT: sacroiliac-leveling exercise; PSIS: posterior superior iliac spine; SI: sacroiliac

At the Canadian Pain Society meeting in April 2018, I presented a poster of a chart review of all my low back pain patients treated between 2015 and 2017 using SIFFT directed leveling exercises (SIFFT-E). I called the poster “how to diagnose and treat low back pain in less than five minutes with >70% chance of relieving the pain.” It was a review of 180 charts between 2015 and 2017, where I had recorded the diagnosis of low back pain, lumbar spondylosis, lumbar disc herniation, sciatica, or sacroiliac sprain. The affected patients averaged 61.8 years of age (between 20 and 90 years), 106 women and 58 men, and averaged three years of low back pain. Only 16 (9%) patients had level sacroiliac joints, indicating their pain was probably lumbar in origin. Ninety-one of the 180 (50%) patients had complete and 50 (28%) had partial relief, following the two-minute corrective exercise which repositioned the affected sacroiliac joint (Figure 1).

Before I retired at the age of 77 years, I conducted a randomized controlled study comparing the effectiveness of conventional treatments with that of the pelvic stabilization belt and the SIFFT-E. Ninety percent of those with a displaced sacroiliac joint experienced relief after doing the two-minute SIFFT-E. I gave my patients a short YouTube video that allowed them to locate the PSISs themselves and taught them what corrective exercise they needed to perform (Video 1) [4]. The below video was created to help patients with unstable sacroiliac joints determine the relative position of the affected joint and the corresponding corrective exercise.

**Video 1:** Short instructional video on how to diagnose a sacroiliac joint displacement and use a two-minute exercise to correct it.

Many of my patients found that performing the exercise relieved their chronic back pain, and they were happy to be able to treat themselves at home without needing additional tests or treatments. Wearing a pelvic stabilization belt helped prevent further sacroiliac (SI) displacement [5].

In April 2011, at the AAOM meeting, Dr. John Lyftogt from New Zealand gave a one-day seminar attended by about 50 physicians, in which he described how quickly and effectively perineural injections of a buffered solution of 5% dextrose provided pain relief [6]. We were understandably skeptical until, at the end of the seminar, he asked for volunteers with pain to be treated with this procedure. Seven attendees with a variety of painful conditions from headaches to neck, shoulder, back, knee, and, foot pain raised their hands. He treated them and they all left the stage entirely pain-free. At this seminar by Dr. Lyftogt, I discovered the safety and effectiveness of 5% dextrose perineural injections to provide immediate pain relief to any area supplied by a nerve I could reach with my needle [6].

I started using this technique on my patients, with very good results. I would first identify painful nerves through palpation and using ultrasounds, which allowed me to visualize the needle as it approached the nerve. At times, I could see the solution release the pain-producing nerve from scar tissue that had tightened around it. The relief these patients felt would then last weeks and repeat injections produced longer and longer responses until there was no more pain. As I was treating many diabetics with peripheral neuropathy, I shifted my perineural injection material to 5% mannitol, which may be as effective, with less exposure to dextrose, a potential benefit for diabetics.

Mannitol, a dextrose alcohol, worked just as fast and just as well as the buffered dextrose, and often the pain disappeared even before the needle was out of the skin. Usually, the pain relief lasted between four hours and four days. The more treatments people received, the longer the relief lasted, and the less pain they had.

At that time, Marylene Kyriazis, Pharm.D., a pharmacist who had a special interest in palliative care and pain control, had come to observe my practice. Seeing how effective mannitol was, and knowing most people do not like injections, together we decided that we would try to produce a topical formulation of mannitol to help relieve pain.

We tried this mannitol cream on 235 patients with a total of 289 different painful conditions. I gave them the mannitol cream on the condition that they would return with a questionnaire they would fill out every time they used it. My secretary kept track of who had received the questionnaire and asked for it at their return visit. She downloaded the following information to a database: every time the cream was used, date, time, 0-10 numerical rating scale (NRS) pain score before and 30 minutes after applying the cream, how many minutes to relief, and how long the relief lasted. On average, the cream provided 53% relief in 17 minutes. As some people found complete relief after one application, I could not test the average duration of relief, but the median duration was four hours. We also found that the pain relief was cumulative, the initial pain score decreased with continued application. This was presented as a poster at the Canadian Pain Society meeting on April 3, 2019 [7].

Tess Debelle, a medical student observing my practice, tested the mannitol cream on 20 people with postherpetic neuralgia (PHN), averaging five years duration. It relieved 16 of them. She presented this as a poster on May 24, 2016, at the Canadian Pain Society meeting [8]. She used her results for her bachelor of medicine dissertation at the University of Groningen in the Netherlands.

After 55 years of medical practice, thanks to prolotherapy, the SIFFT-E, and the cream, I am currently pain- and itch-free. At times, I meet people on my daily hikes who suffer from low back pain. Right there, on the forest path, I can examine them and show them how to examine themselves and how to perform the SIFFT-E. After doing the two-minute corrective exercise (which can be done almost anywhere), most of them find pain relief and their pleasure brightens my day. The pain I once considered a curse has brought joy to my life by leading me down a path of discovery that enabled me to find relief, not only for myself but also for many others.

## Discussion

Prolotherapy is the first treatment that brought me relief. Its practitioners need to have an excellent knowledge of musculoskeletal anatomy and the ability to perform accurate injections through palpation or the use of ultrasounds. Given those limitations, it can be performed safely in a family practitioner’s office. Its success rate is high and its side effects, bruising if the needle hits a blood vessel or pain if it hits a nerve, are few and usually not serious, unless treatments are close to the spinal canal.

In 2015, I published a randomized controlled trial (RCT) on prolotherapy for rotator cuff tendinopathy, which showed that this treatment was more effective in relieving pain and improving function than the use of physiotherapy and pain medications [9]. Systematic reviews of this treatment report on its effectiveness [10-12]. Unfortunately, the treatment is not reimbursed by any of the Canadian health insurance systems and is not approved by the FDA, which may be why it is not usually taught in medical school and is neglected by the medical profession.

Dr. Lyftogt who teaches perineural injection therapy (PIT) around the world provided me with my second effective pain treatment [6]. Abdel-Aziz et al. indicate that PIT compares favorably with steroid injections for carpal tunnel syndrome secondary to rheumatoid arthritis [13]. Sallam et al. showed that PIT can be effective in relieving pain and improving function in knee osteoarthritis [14]. Khandelwal and Rath, in a recent narrative review, suggested the perineural injection with buffered 5% dextrose may be helpful in the treatment of trigeminal neuralgia [15]. The treatment is safe, relatively quick and easy to perform, and results in immediate pain relief (even, at times, before the needle is out of the skin). As the results are often cumulative, I believe all physicians who treat people in pain should have instruction in or access to this treatment for their patients.

Developing the mannitol cream was a direct result of doing mannitol-containing perineural injections and provided my third new pain treatment. To date, not many randomized controlled trials (RCTs) have been done on this product. We tested the mannitol cream in an RCT where we applied capsaicin cream to the upper lip of 25 participants to help determine how it provided pain relief and showed that mannitol down-regulates the TRPV1 (capsaicin) receptor [16]. Louw tested the mannitol and menthol cream in a randomized double-blind study comparing it to the same cream without mannitol in treating the symptoms of diabetic peripheral neuropathy. The study has not yet been published.

As the TRPV1 receptor is also essential for the transmission of histamine-induced itch, I am searching for a dermatologist willing to do a randomized placebo-controlled study testing its effectiveness in relieving the itch of psoriasis, eczema, or itchy scalp [17]. I know, from personal experience, it provides immediate relief from the itch and swelling of mosquito bites.

The fourth new pain treatment was forced on me by my own low back pain. As it was resistant to conventional therapy, I had to search for new ways to diagnose and treat it, and I am not alone. In the absence of an accurate diagnosis, a multitude of treatments have been proposed, as outlined by Kirkwood et al., all of which (and others he did not list) I tried and sent my patients to with marginal success [18].

Once I knew how to do the SIFFT-E, it became evident that the most severe low back pain, 86% in my study, is secondary to a displaced sacroiliac joint. I rarely needed to use ultrasound to determine the position of the PSISs as, unless someone was grossly obese or had “back mice” they were easy to locate. The diagnosis was established when doing the two-minute corrective exercise gave the patient immediate pain relief. The SIFFT test and the SIFFT-E are well-defined ways to reach a diagnosis, prescribe a treatment, and measure its effect on the patient [18]. They will allow health practitioners to quickly assess for sacroiliac displacement, if present, correct it, and thus relieve the pain it causes. Of course, the study I did was small and it needs to be replicated with more physicians, who will test intra- and inter-observer reliability, and participants to provide even more statistically significant results.

## Conclusions

Prolotherapy, perineural injection therapy, the SIFFT-E, and topical mannitol are the treatments I learned or discovered while caring for myself or other people in pain, which have provided many with relief. These four techniques address different types of pain: prolotherapy relieves musculoskeletal pain by helping rebuild and strengthens tendons and ligaments, thus stabilizing joints; perineural injection treatments relieve neuropathic pain; the sacroiliac forward flexion test and exercise relieve low back pain from sacroiliac displacement; and the mannitol cream provides relief to structures closer to the skin surface. Together, they give physicians a greater choice of pain-relieving tools for their patients. I hope that those who read this story will avail themselves of these treatments and share their patients' pleasure when they are, at last, free from pain.
